# The expression and functional role of a FOXC1 related mRNA-lncRNA pair in oral squamous cell carcinoma

**DOI:** 10.1007/s11010-014-2093-4

**Published:** 2014-06-03

**Authors:** Xiang-pan Kong, Jie Yao, Wei Luo, Fu-kui Feng, Jun-tao Ma, Yi-peng Ren, De-li Wang, Rong-fa Bu

**Affiliations:** 1 Department of Stomatology, Chinese PLA General Hospital, Chinese PLA Medical School, Beijing, 10086 China; 2 Department of Periodontics, Tianjin Stomatology Hospital, Nan Kai University, 300041 Tianjin, China; 3 Department of Oncology, PLA No. 161 Center Hospital, Wuhan, Hubei China; 4 The First Affiliated Hospital of Dalian Medical University, Dalian, Liaoning China; 5 MuDanJiang Medical College, Mudanjiang, Heilongjiang China

**Keywords:** LncRNA, FOXC1, Oral squamous cell carcinoma, Proliferation, Migration

## Abstract

The Fork head box C1 (FOXC1) gene is overexpressed in multiple malignant tumors and is functionally correlated with tumor progression. However, its’ role in oral squamous cell carcinoma (OSCC) is still unclear. Recent studies have revealed that many long non-coding RNA (lncRNAs) cooperate with adjacent coding genes and form a functional “lncRNA-mRNA pair”. In this study, we report a new lncRNA FOXC1 upstream transcript (FOXCUT) that was remarkably overexpressed in 23 OSCC patients, as was the adjacent FOXC1 gene. The expressions of FOXC1 and FOXCUT were positively correlated. When the expression of FOXCUT was down-regulated by small interfering RNA (siRNA), the expression of FOXC1 was also decreased. Moreover, in OSCC cells Tca8113 and SCC-9, down-regulation of either FOXC1 or FOXCUT by siRNA could inhibit cell proliferation and cell migration in vitro and was accompanied with a reduction of MMP2, MMP7, MMP9, and VEGF-A. In conclusion, FOXC1 may be co-amplified with FOXCUT in OSCC, and both of them may be functionally involved in the tumor progression of OSCC. This provides evidence that both FOXC1 and FOXCUT may serve as novel biomarkers and therapeutic targets in OSCC patients who overexpress this “lncRNA-mRNA pair”.

## Introduction

Oral squamous cell carcinoma (OSCC) is one of the most aggressive neoplasms among head and neck malignant tumors [1]. Despite considerable recent advances in treatment, the prognosis of advanced OSCC remains poor. Many studies have revealed multiple genetic, multistep changes in the development of the OSCC [2, 3]. Therefore, a better comprehension of the molecular mechanisms underlying OSCC is necessary for selecting suitable predictive biomarkers and seeking emerging tailored therapeutic strategies.

The fork head box C1 (FOXC1) gene, a member of the FOX family, is located on chromosome 6p25 [4] and is a transcriptional factor that regulates a wide array of biological processes including the maintenance of differentiated cell states, embryogenesis, tumorigenesis, and tumor progression, such as epithelial-mesenchymal transition (EMT) and cell migration [5–7]. Several recent studies have reported that FOXC1 overexpression is strongly correlated with poor survival in the patients of multiple cancers such as breast cancer, pancreatic ductal adenocarcinoma, non-small cell lung cancer, or hepatocellular carcinoma [6–11]. However, overexpression of FOXC1 has not been reported in OSCC yet.

Because transcriptional factors normally do not act alone, it is likely that FOXC1 is a component of a larger complex that regulates the initiation of transcription, and it is important to find the molecules that interact with FOXC1 to impact tumorigenesis and tumor progression. Lately, transcriptome wide analysis has shown that >90 % of the human genomic DNA is transcribed into non-coding RNAs (ncRNAs) that lack protein-coding potential. Among those, long non-coding RNAs (lncRNAs) are those ncRNA transcripts ranging from 200 bases to 100 kilo bases (kb) in length [12–15]. In malignant tumors, numerous unique lncRNA molecules have been found that are closely associated with the development and progression of a variety of cancers [14].

Recent studies have revealed that a large proportion of lncRNAs cooperate with adjacent protein-coding genes and form “lncRNA-mRNA pairs” that impact their function. Close relationships are often found between these lncRNAs and their nearby mRNAs in expression or function. The “lncRNA-mRNA” pair is now regarded as a new form in the very complex gene expression modulating network. [16].

Through bioinformatic analysis, we found an lncRNA transcribed from the upstream region of FOXC1 promoter that we named FOXC1 upstream transcript, or, FOXCUT. We speculated that the divergent transcription of this lncRNA-mRNA pair (FOXC1 and FOXCUT) may play an important role in OSCC.

In this study, we investigated the expression pattern of FOXC1 and lncRNA-FOXCUT in OSCC tissues, the correlation between the expression of this lncRNA-mRNA pair, and the functional role of this pair in OSCC in vitro. The aim of this study was to possibly identify a new functional lncRNA-mRNA pair involved in OSCC.

## Materials and methods

### Patient samples

A total of 23 fresh OSCC tissue specimens together with matched adjacent nontumorous tissue specimens were collected from patients who underwent surgery in our Hospital, China, between 2012 and 2013. The OSCC diagnosis was histopathologically confirmed. None of the patients received preoperative therapy before surgical resection. All tissue specimens were immediately frozen and stored in liquid nitrogen after surgery until the extraction of total RNA. This research was approved by the ethical committee of PLA General Hospital. The procedures followed were in accordance with the ethical standards established by the Ethics Committee of our Hospital.

### Cell line and cell culture

The human OSCC cell lines used were Tca8113, OSC-4, SCC1, SCC2, SCC4, SCC9, CAL-27, UM1, and UM2. The human hepatocellular carcinoma cell lines used were SMMC7721, HCCLM3, HUH7, and HEPG2. The human breast cancer cell lines used were MCF7, BT474, SKBR3, HCC1187, and HCC1143. The normal human liver cell line LO2, the normal human oral keratinocyte cell line HOK, and the normal human breast cell MCF-10A were purchased from American type culture collection (Manassas, VA, USA). The Tca8113, CAL-27, MCF 10A, SMMC7721, HCCLM3, HEPG2, and BT474 cells were incubated in DMEM medium (Hyclone, Logan, UT) containing 10 % FBS (Invitrogen, Carlsbad, CA) at 37 °C with 5 % CO2. The OSC-4, SCC2, SCC9, LO2, HCC1143, and SKBR3 cells were incubated in PRMI-1640 (Gibco, American) containing 10 % FBS. The SCC-4, MCF7, and HUH7 cells were incubated in DMEM: F12 containing 10 % FBS. The HOK cells were incubated in OKM containing growth factor. Approximately 5 % of Tca8113 cells were plated into each well of 12-well plates at least 24 h before transfection to achieve 30–50 % confluency.

### Quantitative PCR

Total RNA was extracted from OSCC tumor tissues, matched adjacent normal tissues and OSCC cells using trizol total RNA reagent (Invitrogen, Carlsbad CA). Primers were obtained from Sheng Gong (Shanghai, China), and their sequences are shown in Table 1. Quantitative PCR was performed using the SYBR primescript RT-PCR kit (Takara, Ohtsu, Japan) in an Applied Biosystems 7500 Fluorescent Quantitative PCR System (Applied Biosystems, Foster City, CA). The reaction mixtures were incubated at 95 °C for 30 s, followed by 40 amplification cycles of 95 °C for 5 s and 60 °C for 34 s. The comparative Ct method was used to quantify relative expression of mRNA and lncRNA. Expression level of the housekeeping gene, β-actin, was used to normalize gene-of-interest expression. The expression level of each target gene in a patient was calculated as the ratio of target in tumor tissue/target in nontumorous tissue [R (T/N)].

**Table 1:** Primers for real-time PCR analysis

Genename	Forward (5′–3′)	Reverse (5′–3′)
β-actin	CCACTGGCATCGTGATGGA	CGCTCGGTGAGGATCTTCAT
FOXC1	GGCGAGCAGAGCTACTACC	TGCGAGTACACGCTCATGG
FOXCUT	GTCGCACCGATGACTAACG	GCCCTGAAAGCCGAACTG
MMP1	GGGAGATCATCGGGACAACTC	GGGCCTGGTTGAAAAGCAT^[]^
MMP2	CTGACCCCCAGTCCTATCTGCC	TGTTGGGAACGCCTGACTTCAG
MMP3	CTGGACTCCGACACTCTGGA	CAGGAAAGGTTCTGAAGTGACC
MMP7	GAGATGCTCACTTCGATGAGG	GGATCAGAGGAATGTCCCATAC
MMP-9	CTTTGACAGCGACAAGAAGTGG	GGCACTGAGGAATGATCTAAGC
MMP-13	TTGTTGCTGCGCATGAGTTCG	GGGTGCTCATATGCAGCATCA
VEGF-A	AGGGCAGAATCATCACGAAGT	AGGGTCTCGATTGGATGGCA

### Western blot assay

Cells were washed twice with chilled PBS and lysed directly in wells by incubating with RIPA lysis buffer supplemented with protease inhibitor (Roche) 48 h post-transfection. Equal amounts of protein were size fractionated by SDS-PAGE on 4–12 % Bis–Tris acrylamide NuPAGE gels in MOPS SDS running buffer (Invitrogen), and transferred onto a nitrocellulose membranes (Whatman) in NuPAGE transfer buffer (Invitrogen) containing 10 % methanol. Membranes were blocked with 5 % skimmed milk at room temperature for 2 h, incubated overnight (4 °C) with primary antibodies, and then incubated with corresponding secondary antibody for 60 min at room temperature.

### Transfection of siRNA

The siRNA sequences were obtained from gene pharma (Shanghai, China), including one negative control siRNA(NC siRNA) sequence, two lncRNA-FOXCUT siRNA sequences, and two FOXC1 siRNA sequences. The target sequences are shown in Table 2. siRNA transfection was performed with X-treme GENE transfection reagent (Roche) according to the manufacturer’s instructions. Approximately 5 % Tca8113 and SCC-9 cells were plated into each well of 12-well plates at least 24 h before transfection to achieve 30–50 % confluency. After two washes with PBS, the Tca8113 cells in each group were transfected by NC siRNA, FOXCUT siRNAs (si1, si2), or FOXC1 siRNAs (si1, si2) for at least 48 h. After transfection, total cells were collected for RNA isolation, cell proliferation assay, colony formation assay, and scratch wound healing assay.

**Table 2:** Target sequences for small interfering RNA analysis

Genename	Sense strand	Antisense strand
FOXC1 si 1	5′rGrCrArGrUrArArUrUrGrCrUrGrUrUrGrCrUrUrGrUrUrGTC	5′rGrArCrArArCrArArGrCrArArArCrArGrCrArArUrUrArCrUrGrCrUrU
FOXC1 si 2	5′rCrGrUrUrArArArUrUrGrCrCrUrGrArArArCrUrUrUrArAAT	5′rArUrUrUrArArArGrUrUrUrCrArGrGrCrArArUrUrUrArArCrGrUrC
FOXCUT si1	5′rGrArArUrGrGrArGrArArCrUrArArGrArCrArArUrUrArUCT	5′rArGrArUrArArUrUrGrUrCrUrUrArGrUrUrCrUrCrCrArUrUrCrGrG
FOXCUT si2	5′rGrArGrGrGrArCrUrGrUrGrCrUrGrArCrArArArUrCrCrUCT	5′rArGrArGrGrArUrUrUrGrUrCrArGrCrArCrArGrUrCrCrCrUrCrCrA

### Cell proliferation assay

Cells proliferation was measured by MTS according to the manufacturer’s protocol. In brief, after transfection, the Tca8113 cells (2,000cells/well) were seeded into 96-well plates. The cells were incubated for 0, 1, 2, or 3 days, Then, 20 μl of MTS reagent (Promega) was added to each well containing 100 μl culture medium, and the plates were incubated routinely for 1 h at 37 °C. The absorbance values of each well were measured with a universal microplate reader at a wavelength of 490 nm.

### Colony formation assay

Cells were trypsinized, counted, and seeded at a low density of 600 cells/well on six-well culture plates. Cells grew undisturbed at 37 °C in 5 % CO_2_ for 2 weeks and then stained with crystal violet.

### Scratch wound healing assay

Uniform wounds were made in Tca8113 epithelial monolayers grown on plastic 12-well plates using a pipette tip before transfection. Microphotographs were taken at 0 h, 24 h, and 48 h after siRNA transfection.

### Statistical analysis

Differences between groups were analyzed using Student’s *t* test. Correlation between gene expression ratios was studied by using Pearson’s correlation. Statistical analyses were performed by using SPSS version 18.0 (SPSS, Chicago, IL) and GraphPad Prism Software (GraphPad Software, Inc., San Diego, CA). For all statistical analyses, *p* < 0.05 was considered statistically significant.

## Results

### FOXC1 and FOXCUT were co-overexpressed in OSCC tissue specimens and OSCC cell lines

**Fig. 1:**
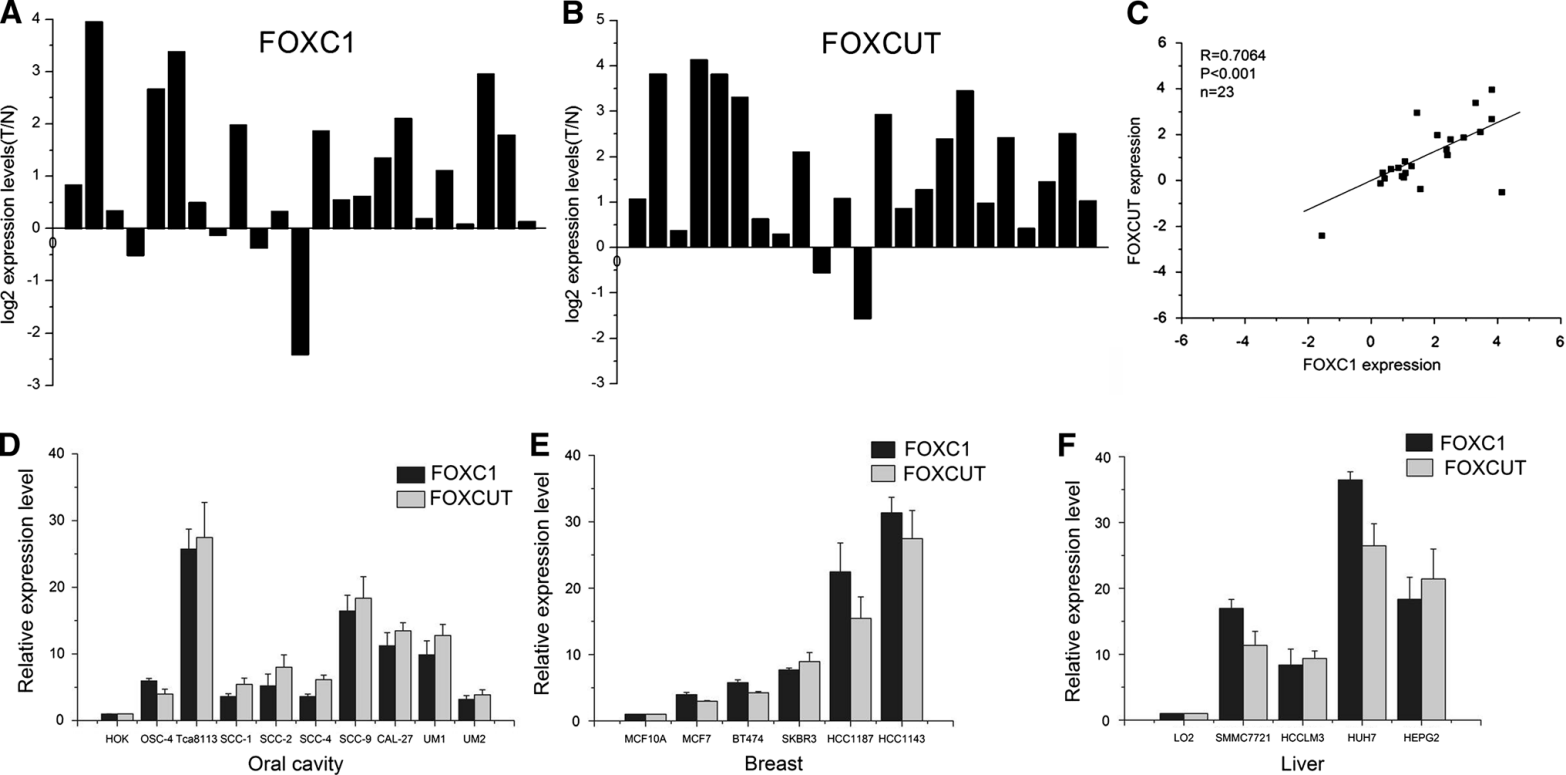
The lncRNA-FOXCUT and FOXC1 expression levels were analyzed by real-time PCR in 23 OSCC tissue samples, 9 human OSCC cell lines Tca8113,OSC-4, SCC1, SCC2, SCC4, SCC9, CAL-27, UM1, and UM2; 4 human hepatocellular carcinoma cell lines, SMMC7721, HCCLM3, HUH7, and HEPG2; 5 human breast cancer cell lines MCF7, BT474, SKBR3, HCC1187, and HCC1143; and normal human liver cells LO2, normal human oral keratinocyte cell line HOK, normal human breast cell MCF-10A. **a** The expression levels of FOXC1 in OSCC were significantly higher than those in adjacent normal tissues by Real-Time Quantitative PCR, **b** the expression levels of lncRNA FOXCUT were also higher than those in adjacent normal tissues by Real-Time Quantitative PCR. **c** The expression level of FOXC1 was significantly correlated with that of lncRNA-FOXCUT (*R* = 0.7063 *p* < 0.0001), **d** the expression levels of FOXC1 mRNA and FOXCUT lncRNA were apparently elevated in all the nine OSCC cell lines compared with normal human oral keratinocyte cell line HOK. **e**, **f** additionally, the FOXC1 and FOXCUT were co-overexpressed in all the hepatocellular carcinoma cell lines and breast cancer cell lines than in the normal cells (* indicates *p* < 0.05)

The FOXC1 mRNA and FOXCUT lncRNA expression levels were assessed in a group of 23 patients with OSCC and 23 corresponding adjacent normal tissues by Real-Time Quantitative PCR. We found that 19 of the 23 OSCC patients (82.6 %, *p* < 0.05) showed remarkably higher expression of FOXC1 mRNA in tumor tissues than in noncancerous tissues (Fig. 1a). In addition 21 of the 23 patients (91.3 %, *p* < 0.05) showed remarkably higher expression of FOXCUT lncRNA in tumor tissues than in noncancerous tissues (Fig. 1b). The relative expression of FOXC1 positively correlated with that of FOXCUT in OSCC tissue samples. The expression levels of FOXC1 mRNA and FOXCUT lncRNA were then evaluated in a panel of OSCC cell lines, Tca8113, OSC-4, SCC1, SCC2, SCC4, SCC9, CAL-27, UM1, UM2, and a normal human oral keratinocyte cell line, HOK. The results showed that FOXC1 and FOXCUT were both overexpressed in all nine of the OSCC cell lines compared with the normal human oral keratinocyte cell line HOK. To further confirm these results, we also investigated the expression of FOXC1 and FOXCUT in four hepatocellular cancer cell lines and five breast cancer cell lines, and found that both FOXC1 and FOXCUT expressions were higher in these cancer cells than in normal cells. Moreover, among the nine OSCC cells lines, Tca8113 and SCC-9 cells displayed the most apparent FOXC1 and FOXCUT dual overexpression. Therefore, we chose Tca8113 and SCC-9 as the candidate cells in the FOXC1 and FOXCUT knock-down experiments.

### FOXC1 expression in Tca8113 cells was suppressed by FOXC1 siRNA and FOXCUT siRNA

**Fig. 2:**
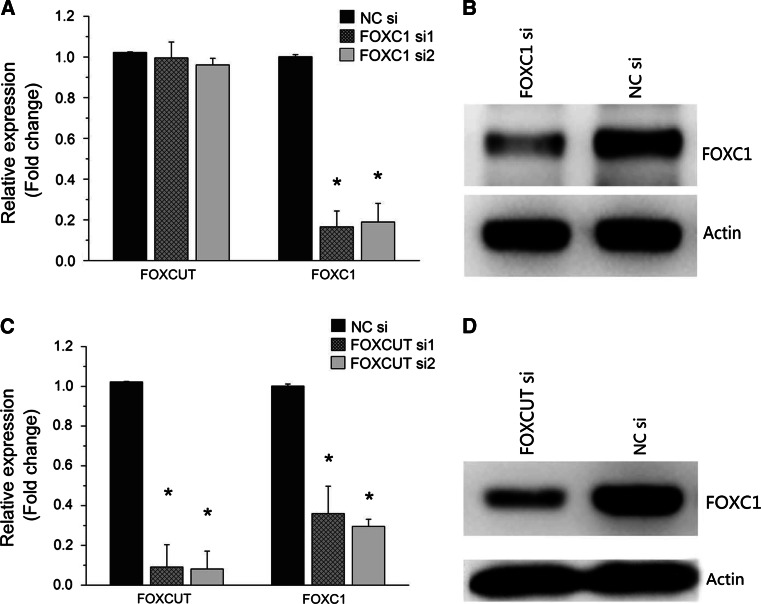
The expression levels of FOXC1 mRNA and FOXCUT lncRNA in Tca8113 cells after siRNA transfection. **a** The expression levels of FOXC1 in FOXC1 siRNA group were significantly knocked down in Tca8113 cells (* indicates *p* < 0.05), but the expression lncRNA FOXCUT did not decrease. **b** FOXC1 protein expression also markedly decreased in FOXC1 siRNA group. **c** The expression levels of both FOXCUT and FOXC1 were significantly knocked down in FOXCUT siRNA group (* indicates *p* < 0.05). **d** FOXC1 protein expression also markedly decreased in FOXCUT siRNA group

In Tca8113 cells, RNAi analysis was conducted to further clarify the correlation between the expression of FOXCUT and FOXC1. Real-time PCR was performed to evaluate the expression of FOXC1 mRNA and FOXCUT lncRNA, and western blot was performed to assess the expression of FOXC1 protein. The results showed that the FOXC1 expression level was down-regulated in FOXC1 siRNA groups compared with NC siRNA group (Fig. 2a, b). Both FOXC1 siRNA1 and FOXC1 siRNA2 knockdown were efficient; they were down-regulated by nearly 80 % (Fig. 2a).

Moreover, the FOXC1 expression levels were also down-regulated in FOXCUT siRNA groups compared with the NC siRNA group (Fig. 2c). The FOXCUT lncRNA expression was decreased by almost 90 %, and the FOXC1 mRNA expression was also suppressed by approximately 70 % (Fig. 2c). The expression of FOXC1 protein was also decreased, which was certified by western blot (Fig. 2d).

However, given that the FOXC1 expression was markedly reduced by approximately 80 % in FOXC1 siRNA groups (Fig. 2a), the FOXCUT expression levels did not decrease together (Fig. 2a).

### Knockdown of FOXC1 inhibited the cell proliferation and migration ability in Tca8113 and SCC-9

To investigate the effects of FOXC1 knockdown on the in vitro growth characteristics of the OSCC cell lines Tca8113 and SCC-9, MTS and colony formation assays were employed to assess cell proliferation ability, and a scratch wound healing assay was performed to evaluate cell migration ability.

**Fig. 3:**
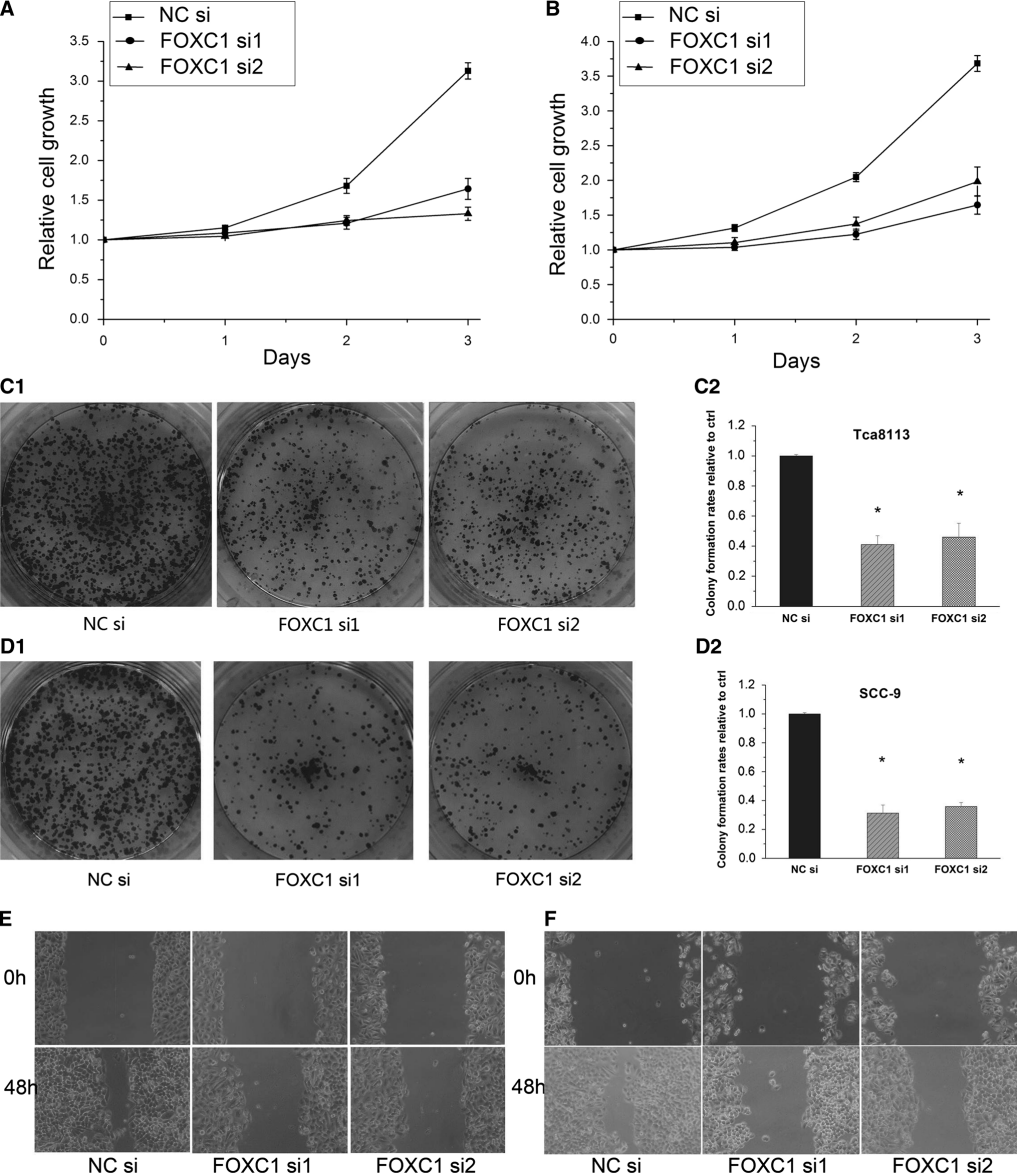
Knockdown of FOXC1 inhibited the cell proliferation and migration ability in Tca8113 and SCC-9. **a**, **b** FOXC1 siRNAs reduced the Tca8113 and SCC-9 cells growth compared with NC siRNA in MTS assay. **c**, **d** The colony formation rates of Tca8113 and SCC-9 cells were significantly decreased by FOXC1 siRNAs (*p* < 0.01). **e**, **f** The capacity of cell migration in FOXC1 siRNAs groups was remarkably impaired

The MTS results showed that cell proliferation was inhibited in the FOXC1 siRNA groups (Fig. 3a, b, *p* < 0.05), and the number of colonies was also significantly decreased in FOXC1 siRNA groups compared to the NC siRNA group (Fig. 3c, d, *p* < 0.05). The scratch wound healing assay results showed that the cell migration was also retarded by FOXC1 siRNAs (Fig. 3e, f).

### Knockdown of FOXCUT inhibited the cell proliferation and migration ability in Tca8113 and SCC-9

**Fig. 4:**
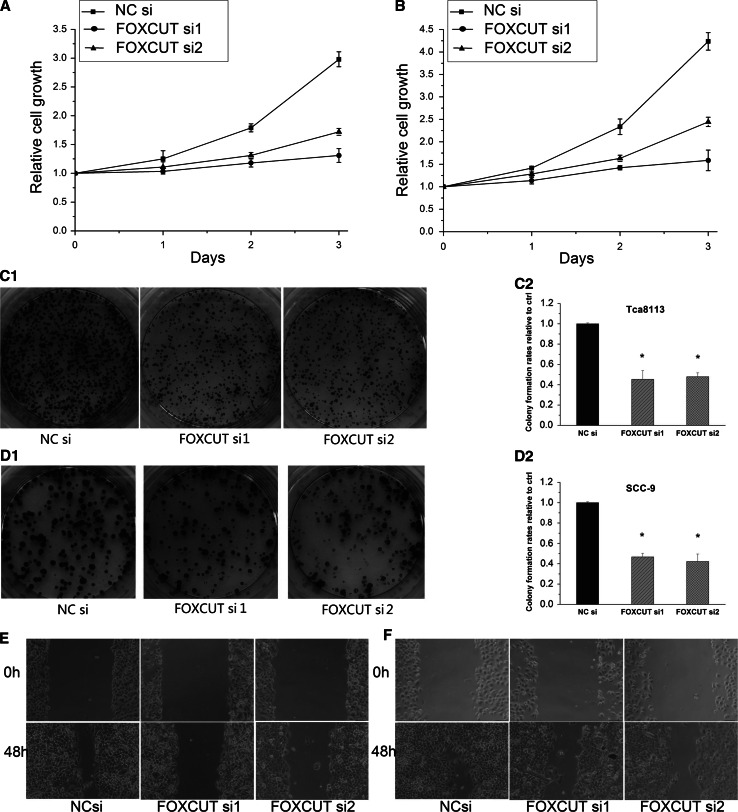
Knockdown of FOXCUT inhibited the cell proliferation and migration ability in Tca8113 and SCC-9. **a**, **b** FOXCUT siRNAs reduced the Tca8113 and SCC-9 cells growth compared with NC siRNA in MTS assay. **c**, **d** The colony formation rates of Tca8113 and SCC-9 cells were significantly decreased by FOXCUT siRNAs (*p* < 0.01). **e**, **f** The capacity of cell migration in FOXCUT siRNA groups was impaired

To further identify the function of FOXCUT, we also performed an MTS assay, a colony formation assay and a scratch wound healing assay after siRNA transfection. The results showed that the growth ability of the Tca8113 and SCC-9 cells was significantly inhibited by FOXCUT siRNA1 (Fig. 4a, b, c, d, *p* < 0.05), and the cell migration capacity was also retarded (Fig. 4e, f). These functional test results following FOXCUT knockdown were in agreement with the effects of FOXC1 knockdown on the in vitro growth characteristics of Tca8113 and SCC-9 as shown above.

### Knockdown of FOXC1 and FOXCUT reduced the expression of certain MMPs and VEGF-A

**Fig. 5:**
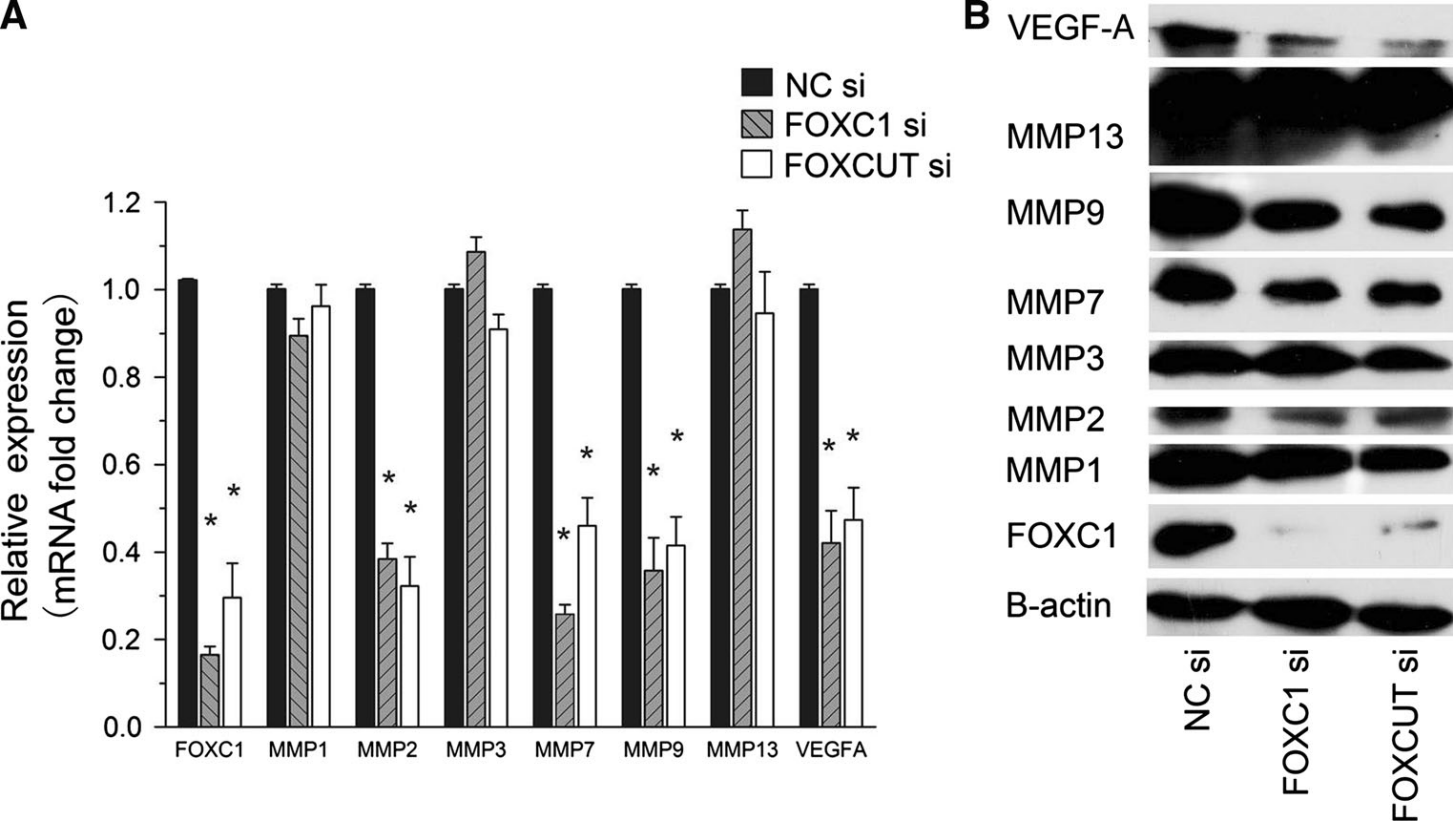
Effect of FOXC1 and FOXCUT knockdown on the expression of certain MMPs and VEGF-A. **a**, **b** Real-time PCR and Western blot examine showed that the expressions of MMP2, MMP7, MMP9, and VEGF-A were apparently decreased together with FOXC1 and FOXCUT knocking down in the Tca8113 cell after transfection

The matrix metalloproteinases (MMPs) have been identified as important indicators in OSCC cell migration [8], [17]. Furthermore, MMP expressions, as well as some angiogenesis factors such as VEGF-A, have correlated with FOXC1 expression in cancers [7]. These findings prompted us to evaluate the effect of FOXC1 and FOXCUT knockdown on MMPs and VEGF-A expressions. The results of real-time PCR and western blot showed that the expression of MMP2, MMP7, and MMP9 were decreased concomitantly with FOXC1 and FOXCUT knocking down (Fig. 5a, b). In contrast, the expression of MMP1, MMP3, and MMP13 were not affected by FOXC1 and FOXCUT silencing. Additionally, the results also confirmed that the expression level of angiogenesis factor VEGF-A was reduced after FOXC1 and FOXCUT knockdown (Fig. 5a, b), which may indicate a possible role for FOXC1 and FOXCUT in tumor angiogenesis.

## Discussion

OSCC is a complicated and multi-step malignant tumor that results from a multitude of genetic mutations. Genetic disorders in OSCC have been widely studied, and an increasing number of tumor suppressor genes and oncogenes have been identified that contribute to OSCC [18].

Previous investigations about OSCC-related genes are mainly focused on protein-coding genes. Recently, an emerging role for lncRNAs in various cancers has been identified in an increasing number of studies. [14]. However, the expression and functional role of most lncRNAs are still unknown in the disease progression of OSCC.

In this study, we first identified a new lncRNA-mRNA pair, FOXC1 and its adjacent lncRNA FOXCUT, as the new form of cancer driver gene compound in OSCC.

FOXC1 was shown to be involved in the occurrence and development of various tumors by affecting the cell proliferation, epithelial-to-mesenchymal transition (EMT), and cell migration [6, 19]. However, in OSCC, the role of FOXC1 has not yet been identified. The function of FOXC1 and the related possible regulatory mechanisms in OSCC is necessary to be clarified.

FOXC1 upstream transcript (FOXCUT) is a FOXC1 adjacent lncRNA which belongs to a class of ncRNAs called promoter upstream transcripts (PROMPTs) [20–22]. The expression and function of PROMPTs are often associated with the adjacent protein coding transcripts. Therefore, we speculated that lncRNA-FOXCUT may regulate the cancer cell growth characteristics of OSCC by regulating FOXC1.

Based on these finding, we investigated the expression pattern of the lncRNA/mRNA gene pairs of lncRNA-FOXCUT and mRNA-FOXC1 in 23 OSCC tissue samples by real-time PCR. The results showed that the expression level of lncRNA-FOXCUT was positively correlated with FOXC1 mRNA. When the expression of FOXCUT was down-regulated by siRNA, the expression of FOXC1 was also decreased. However, the down-regulation of FOXC1 did not affect the expression of lncRNA FOXCUT. It suggests that the lncRNA-FOXCUT may be a regulator of FOXC1 gene expression. However, lncRNAs can regulate adjacent protein-coding gene activity by many different ways, and the specific molecular mechanisms underlying the regulatory impact of this lncRNA-mRNA pair has not been described in this study.

The real-time PCR results also showed that both the lncRNA-FOXCUT expression and the mRNA FOXC1 expression were significantly elevated in OSCC tumor tissues and OSCC cell lines compared with normal oral cavity tissues and oral keratinocyte cell. For the first time, we revealed that both the RNA of FOXCUT and FOXC1 can be stably detected, and both may serve as novel molecular markers for OSCC.

In addition, we further explored a possible functional role of this lncRNA-mRNA pair in OSCC cell growth characteristics using siRNA. As mentioned above, in many other malignant tumors, FOXC1 has been demonstrated to be a cancer-related factor involved in tumor progression processes such as cell proliferation and cell migration. Therefore, we assessed the cell proliferation and migration ability after FOXCUT/FOXC1 siRNA transfection by using MTS, colony formation assay and scratch wound healing assay. The results showed that the knockdown of either FOXCUT or FOXC1by siRNA could inhibit the proliferation and migration of OSCC cell Tca8113 and SCC-9.

MMPs are set of critical factors in the migration and invasion of OSCC cells [17]. Expression of some MMPs correlated with FOXC1 expression, such as MMP7 in breast cancer [8] and MMP1, MMP2, MMP7, and MMP9 in hepatocellular cancer [7]. We hypothesized that these MMPs may also be associated with the FOXC1 and FOXCUT gene pair. Therefore, we determined the expression of these MMPs by both mRNA and protein levels after FOXC1 and FOXCUT down-regulation. In accordance with our hypothesis, MMP2, MMP7, and MMP9 expressions decreased together following both FOXC1 and FOXCUT knockdown, which indicate that these four MMPs could be involved in FOXC1/FOXCUT gene-pair related function in OSCC cell migration.

EMT has been demonstrated to promote angiogenesis by modulating factors such as VEGF-A [7]. Because FOXC1 is an EMT-related gene, and the relationship between FOXC1 and VEGF-A has also been proved in hepatocellular cancer [7], here, we also revealed that the knockdown of FOXC1/FOXCUT gene pair could lead to VEGF-A down-regulation. This may suggest a possible role for the FOXC1/FOXCUT gene pair in tumor angiogenesis in OSCC.

In conclusion, these results suggest that in the lncRNA-mRNA pair, FOXCUT and FOXC1 are both novel overexpressed functional molecules in OSCC. This study is the first to identify the expression and functional role of an lncRNA-mRNA pair in OSCC. FOXCUT and FOXC1 could be represent the potential diagnostic markers and therapeutic targets in the cancer clinic.
